# Single cell transcriptome sequencing of inspiratory neurons of the preBötzinger complex in neonatal mice

**DOI:** 10.1038/s41597-022-01569-y

**Published:** 2022-07-30

**Authors:** Caroline K. David, Yae K. Sugimura, Prajkta S. Kallurkar, Maria Cristina D. Picardo, Margaret S. Saha, Gregory D. Conradi Smith, Christopher A. Del Negro

**Affiliations:** 1 Department of Applied Science, William & Mary, 540 Landrum Drive, Williamsburg, Virginia 23185 USA; 2 Department of Neuroscience, Jikei University School of Medicine, 3-25-8 Nishi-shimbashi, Minato, Tokyo, 105-8461 Japan; 3 Department of Biology, William & Mary, 540 Landrum Drive, Williamsburg, Virginia 23185 USA

**Keywords:** Gene expression profiling, Transcriptomics

## Abstract

Neurons in the brainstem preBötzinger complex (preBötC) generate the rhythm and rudimentary motor pattern for inspiratory breathing movements. We performed whole-cell patch-clamp recordings from inspiratory neurons in the preBötC of neonatal mouse slices that retain breathing-related rhythmicity *in vitro*. We classified neurons based on their electrophysiological properties and genetic background, and then aspirated their cellular contents for single-cell RNA sequencing (scRNA-seq). This data set provides the raw nucleotide sequences (FASTQ files) and annotated files of nucleotide sequences mapped to the mouse genome (mm10 from Ensembl), which includes the fragment counts, gene lengths, and fragments per kilobase of transcript per million mapped reads (FPKM). These data reflect the transcriptomes of the neurons that generate the rhythm and pattern for inspiratory breathing movements.

## Abstract (summary)

**Table:** 

Measurement(s)	nucleotide sequences
Technology Type(s)	Illumina Sequencing
Factor Type(s)	genotype: Dbx1-derived vs. non-Dbx1-derived neurons in the mouse preBötzinger complex • electrophysiological properties: Type-1 vs. Type-2 inspiratory preBötzinger complex neurons
Sample Characteristic - Organism	Mus musculus
Sample Characteristic - Environment	laboratory environment
Sample Characteristic - Location	contiguous United States of America

## Background & Summary

The inexorable active phase of breathing is inspiration, in which the descent of the diaphragm and elevation of the ribs expands the lungs to draw air in^1^. The rhythm for inspiration emanates from the lower brainstem site called the preBötzinger complex (preBötC)^2–4^. The core rhythmogenic elements of the preBötC are interneurons derived from precursor cells expressing homeobox gene *Developing brain homeobox-1* (*Dbx1*), hereafter referred to as Dbx1 neurons^5–10^. Dbx1 neurons also transmit the inspiratory rhythm as an output pattern to premotoneurons and motoneurons for pump and airway muscles^11,12^. Inspiration begins with a preinspiratory phase attributed solely to rhythmogenic neurons^11,13,14^. When their activity reaches a threshold, an inspiratory burst occurs, which recruits neurons that influence and transmit the pattern for motor output^13–15^. Rhythm- and pattern-generating neurons are the two subsets of the Dbx1 neuron population in the preBötC.

The rhythm- and pattern-generators can be differentiated electrophysiologically. Type-1 neurons are putatively rhythmogenic. They activate with a ramp-like summation of synaptic potentials 300–500 ms prior to the onset of a large-magnitude inspiratory burst^16,17^. Type-1 neurons express A-type transient K^+^ current (*I*_A_). The blockade of *I*_A_ destabilizes the inspiratory rhythm and overall preinspiratory activity *in vitro*^18^. Type-2 neurons are putatively specialized for generating the output pattern. They activate ~300 ms later than Type-1^16,17^ and express hyperpolarization-activated mixed cationic current (*I*_h_), whose manipulation affects motor output^19^.

We differentiated Type-1 and Type-2 neurons electrophysiologically during whole-cell patch-clamp recordings. Despite the well characterized disparity between types, some neurons exhibit ambiguous physiology and cannot be reliably classified; we call those Unknown but nonetheless consider them important for breathing because they are in the preBötC and are rhythmically active in phase with inspiration *in vitro*. We retrieved cytoplasmic contents and performed next-generation RNA sequencing on 10 samples: four Type-1 neurons (three from Dbx1-expressing precursors and one obtained from a CD-1 wild-type mouse without a Dbx1 reporter), one Type-2 neuron from a Dbx1-expressing precursor, as well as 5 Unknown neurons from CD-1 wild-type mice. All the neurons were recorded and RNA extracted within two months and sent for next-generation sequencing together.

## Methods

### Mice

The Institutional Animal Care and Use Committee at William & Mary approved these protocols, which conform to the policies of the Office of Laboratory Animal Welfare (National Institutes of Health, Bethesda, MD, USA) and the guidelines of the National Research Council^20^. CD-1 mice (Charles River, Wilmington, MA) and genetically modified mice (described below) were maintained on a 14-hour light/10-hour dark cycle at 23 °C and were fed *ad libitum* with free access to water.

Experiments employed CD-1 mice or an intersectional mouse model dubbed Dbx1;Ai148. Regarding nomenclature for the intersectional model, the first part refers to Dbx1^Cre^ mice (IMSR Cat# EM:01924, RRID:IMSR_EM:01924) that express Cre recombinase driven by the *Dbx1* promoter^21^. The second part, Ai148 (IMSR Cat# JAX:030328, RRID:IMSR_JAX:030328), refers to Cre-responder mice created by the Allen Brain Institute that express the fluorescent Ca^2+^ indicator GCaMP6f following Cre-mediated recombination of floxed alleles^22^. We crossed Dbx1^Cre^ female mice with Ai148 males to produce offspring with GCaMP6f expression in Dbx1-derived neurons.

### Slice preparations

We cleaned all working spaces with RNase ZAP (Thermo Fisher, Waltham, MA) before each experiment. Tools and glassware were autoclaved or cleaned with RNase ZAP and then rinsed with nuclease-free water (NFW).

We anesthetized CD-1 or Dbx1;Ai148 pups (aged 0–3 postnatal days, i.e., P0-3) by hypothermia as directed by the American Veterinary Medical Association (AVMA, Schaumburg, IL) guidelines version 2020.0.1, which is licensed under the Creative Commons Attribution-Non-Commercial-NoDerivs 3.0. We removed the neuraxis from the pons to the cervical spinal cord in 2 min and placed it into ice-cold artificial cerebrospinal fluid (aCSF) containing (in mM): 124 NaCl, 3 KCl, 1.5 CaCl_2_, 1 MgSO_4_, 25 NaHCO_3_, 0.5 NaH_2_PO_4_, and 30 dextrose. aCSF was prepared ahead of time in an RNase-free environment and then bubbled with 95% O_2_ and 5% CO_2_ throughout the experiments. The neuraxis was trimmed to isolate the brainstem and then glued to the dorsal surface of an agar block, and then positioned in a vise within a vibratome (Campden Instruments 7000 smz-2, Leicester, UK) filled with ice-cold aCSF aerated by 95% O_2_ and 5% CO_2_. We cut a transverse slice 450–500 µm thick with preBötC on its rostral surface^23^. The slice was employed for not more than 3 hours to avoid degradation and contamination of cytoplasmic genetic contents.

### Whole-cell patch-clamp recording and cytoplasmic sample collection

**Fig. 1:**
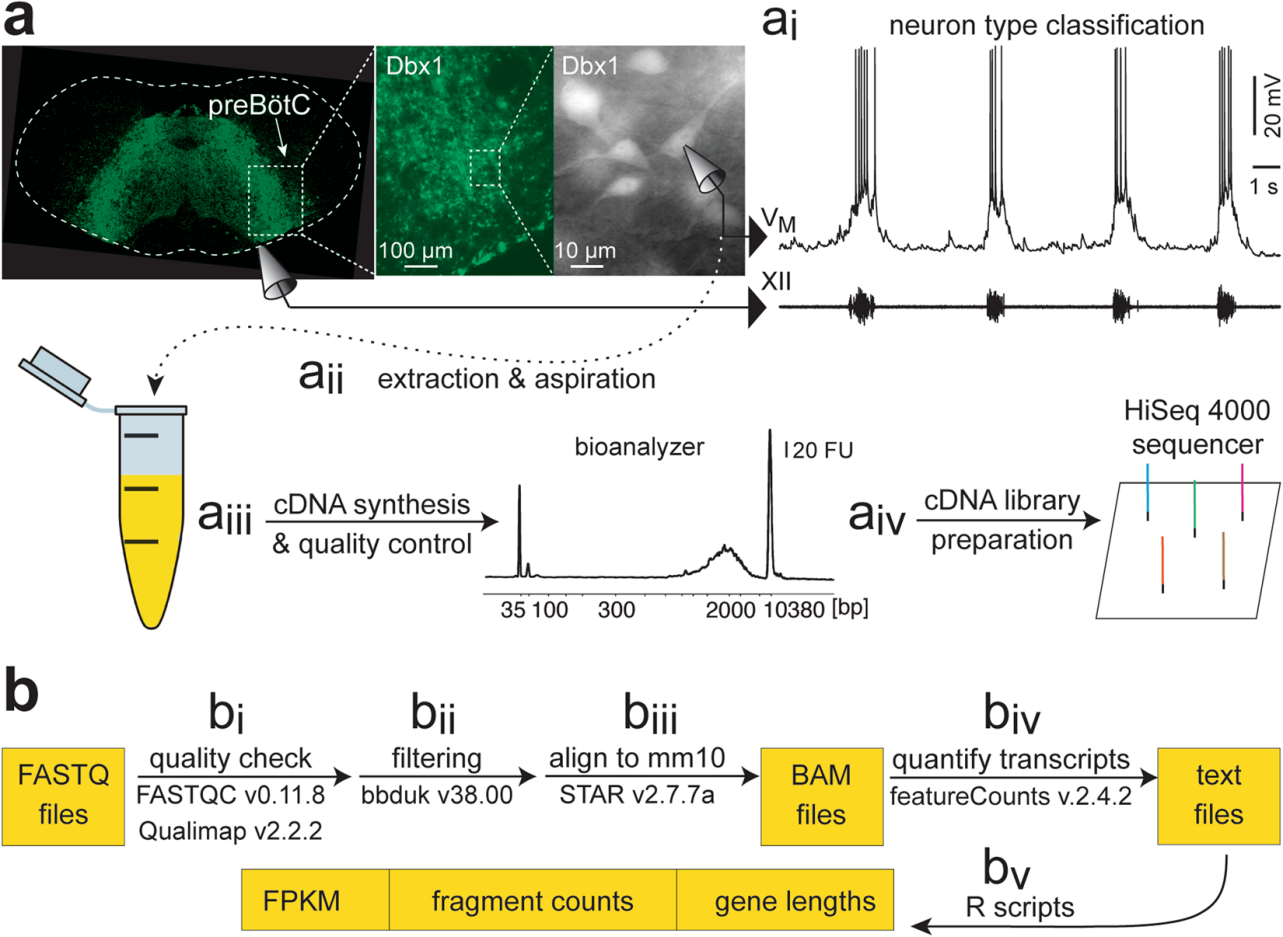
Schematic overview of experimental design. (**a**) The Patch-Seq workflow. Rhythmically active Dbx1-derived preBötC neurons are recorded in the whole-cell patch-clamp configuration (V_M_, top trace). The hypoglossal cranial nerve (XII) represents inspiratory-related motor output (bottom trace). a_i – a_iv graphically represent steps in the Patch-Seq workflow as detailed in Methods. (**b**) Flowchart b_i–b_v showing the bioinformatics workflow as detailed in Methods.

Figure 1a (top) shows our electrophysiology workflow. We perfused slices with aCSF (28 °C) at 2–4 ml/min in a recording chamber on a physiology microscope (Zeiss Axio Examiner, Carl Zeiss AG, Oberkochen, Germany). The external K^+^ concentration of the aCSF was raised from 3 to 9 mM to facilitate robust respiratory rhythm and hypoglossal (XII) motor output^4,24^. We recorded XII motor output using suction electrodes fabricated from autoclaved borosilicate glass pipettes (OD: 1.2 mm, ID: 0.68 mm), initially pulled on a Flaming-Brown P-97 micropipette puller (Sutter Instruments, Novato, CA), then fire polished to a diameter of ~100 µm. XII motor output was amplified by 2,000 using a differential amplifier (EX1, Dagan Instruments, Minneapolis, MN), band-pass filtered at 0.3–1 kHz, and smoothed for display using an analog root-mean-squared filter (Analog Devices, Norwood, MA) to provide a full-wave rectified and smoothed XII waveform. In Dbx1;Ai148 mouse slices we identified inspiratory Dbx1 preBötC neurons based on rhythmic fluorescence changes in sync with XII output before obtaining a whole-cell patch-clamp recording. In CD-1 mice we identified inspiratory preBötC neurons that activated in sync with XII motor output after establishing whole-cell recording. We fabricated patch pipettes from autoclaved borosilicate glass (OD: 1.5 mm, ID: 0.86 mm) using a 4-stage program on the Flaming-Brown P-97 micropipette puller. Tip resistance measured 3–5 MΩ. We filled patch pipettes with internal solution, mixed in an RNase-free environment, containing (in mM): 123 K-gluconate, 12 KCl, 10 HEPES, 0.2 EGTA, 4 Mg-ATP, 0.3 Na-GTP, 10 Na_2_-phosphocreatine, and 13 Glycogen. We adjusted the osmolarity of the internal (patch) solution to 260–270 mOsm and maintained the pH at 7.25. We added 1–2.5% Recombinant Ribonuclease Inhibitor (RRI) (Takara Bio USA, Mountain View, CA) to the internal solution immediately before each experiment to preserve RNA. Robotic micromanipulators (Sensapex, Helsinki, Finland) were employed to position the patch pipettes adjacent to inspiratory neurons under visual control. We performed whole-cell patch-clamp recordings using an EPC-10 patch-clamp amplifier (HEKA Instruments, Holliston, MA) with PATCHMASTER software (RRID:SCR_000034).

### Electrophysiological neuron classification

**Fig. 2:**
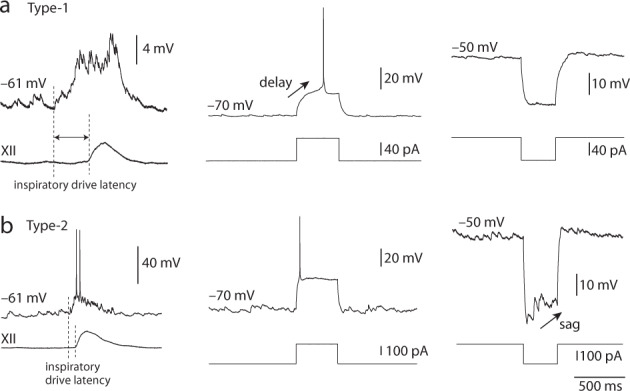
Electrophysiological profiles of preBötC neurons. (**a**) a Type-1 neuron characterized by long inspiratory drive latency (left), delayed excitation (middle), and lack of a ‘sag’ potential (right). (**b**) a Type-2 neuron characterized by short inspiratory drive latency (left), no delayed excitation (middle), and the presence of a sag potential (right).

We defined inspiratory drive latency as the elapsed time between the beginning of summating excitatory synaptic potentials (EPSPs), which depolarize the neuron from its quiescent membrane potential between inspiratory bursts, until the onset of the inspiratory burst. Inspiratory drive latency was used, in part, to determine whether a neuron was Type-1 or Type-2 (Fig. 1ai). Type-1 preBötC neurons show inspiratory drive latency exceeding 300 ms whereas Type-2 preBötC neurons show inspiratory drive latency on the order of 100 ms (compare Fig. 2a, left and b, left).

We tested for A-type K^+^ current (*I*_A_), characteristic of Type-1 preBötC neurons by applying suprathreshold depolarizing current step commands of 500 ms duration from a holding potential of –70 mV. Type-1 preBötC neurons expressing *I*_A_ exhibited delayed excitation of 120–220 ms whereas Type-2 preBötC neurons without *I*_A_ expression do not show delayed excitation in response to similar depolarizing current step commands^16,17,25^ (compare Fig. 2a, middle vs. b, middle).

We tested for hyperpolarization-activated cationic current (*I*_h_) by applying hyperpolarizing current step commands of 400–500 ms duration, which caused initial voltage excursions exceeding −20 mV from a holding potential of –50 mV. Type-2 preBötC neurons expressing *I*_h_ exhibited a time- and voltage-dependent depolarizing ‘sag’ whereas Type-1 preBötC neurons without *I*_h_ expression do not show sag potentials in response to similar hyperpolarizing current step commands^16,17,25^ (compare Fig. 2a, right vs. b, right).

### RNA extraction

Figure 1a (bottom) shows a schematic of our workflow to obtain cytoplasmic contents and create a complementary DNA (cDNA) library suitable for next-generation sequencing. After categorizing the neurons as either Type-1 or Type-2 on basis of inspiratory drive latency and the expression, or lack thereof, of *I*_A_ and *I*_h_; or classifying them as Unknown, we extracted the cellular contents by 0.7–1.5 psi negative pressure (Fig. 1aii). The patch pipette was retracted quickly and the contents of the pipette were ejected by breaking its tip at the bottom of an RNase-free PCR tube containing 4 µL nuclease-free water with 2% RRI. The sample was spun briefly, frozen immediately via submersion in liquid nitrogen and then stored at –80 °C. We converted the extracted RNA to cDNA according to SMART-Seq v4 (Takara Bio USA) ultra-low input RNA kit for sequencing protocol (Fig. 1aiii). First strand cDNA was synthesized by performing reverse transcription in a thermal cycler (42 °C for 90 min, 70 °C for 10 min). The cDNA was then amplified at 95 °C for 1 min, 17 cycles of 98 °C for 10 s, 65 °C for 30 s, 68 °C for 3 min; 72 °C for 10 min. PCR-amplified cDNA was purified by immobilization on AMPure beads (Beckman Coulter, Brea, CA), which we washed with 80% ethanol before eluting the cDNA from the beads. The amplified cDNA was validated using the Agilent 2100 Bioanalyzer and Agilent’s High Sensitivity Kit (5067-4626, Agilent Technologies, Santa Clara, CA). Samples that crossed a threshold of 20 fluorescence units in the range of 1500–2000 base pairs, with little or no peak in the 100–500 base pair region (e.g., Fig. 1a_iii_) were then quantified using Qubit dsDNA High-sensitivity Assay Kit (Molecular Probes, Eugene, OR). Samples with cDNA concentration exceeding 150 pg/µL were retained for next-generation sequencing^26^. We processed full-length cDNA using Nextera Library Preparation Kits (FC-131-1024, Illumina, San Diego, CA) to obtain cDNA libraries for next-generation sequencing using an Illumina HiSeq. 4000 sequencing system with paired end (150 bp) reads (Fig. 1a_iv_). Investigators were blinded to cell type during library construction and sequencing.

### Data processing

Figure 1b shows the schematic for our bioinformatics workflow. First, the quality of reads was verified using FastQC v0.11.8 (Fig. 1b_i_). Adapter reads (Read 1: TCGTCGGCAGCGTCAGATGTGTATAAGAGACAG, Read 2: GTCTCGTGGGCTCGGAGATGTGTATAAGAGACAG) were trimmed using bbduk v38.00 using the following command (Fig. 1b_ii_):


sh bbduk.sh in1 = inputFASTQFile1.fastq.in2 = inputFASTQFile2.fastq out1 = outputFASTQFile1.fastqout2 = outputFASTQFile2.fastq ktrim = r -Xmx27g mm = f k = 33hdist = 1literal = TCGTCGGCAGCGTCAGATGTGTATAAGAGACAG,GTCTCGTGGGCTCGGAGATGTGTATAAGAGACAG tbo tpe


We used the mm10 mouse reference genome from Ensembl (from European Bioinformatics Institute of the European Molecular Biology Laboratory family) to create a genome directory for aligning the reads in STAR^27^ v2.7.7a using the following commands:


STAR --runMode genomeGenerate --genomeDirmm10ReferenceGenome --genomeFastaFilesMus_musculus.GRCm38.dna.primary_assembly.fa --sjdbGTFfileMus_musculus.GRCm38.102.gtf --sjdbOverhang 149 --genomeSAsparseD 2


The sequences were then aligned to mm10 reference genome by STAR v2.7.7a^27^ using the following command (Fig. 1b_iii_):


STAR --genomeDir mm10ReferenceGenome --readFilesIninputFASTQFile1.fastq inputFASTQFile2.fastq --outFileNamePrefix outputBAMFile --outSAMtype BAMSortedByCoordinate --outReadsUnmapped Fastx


We used featureCounts v2.4.2 to quantify fragment counts of the detected genes (Fig. 1b_iv_). Further, we developed custom scripts (see Code Availability, below) in the statistical computing environment “R” to generate text files containing gene lengths, fragment counts, and FPKMs, which are available on NCBI GEO (Fig. 1b_v_).

## Data Records

The raw data consisting of nucleotide sequences (FASTQ files) along with the raw fragment counts of the processed data after alignment to mm10 (GSE175643_fragmentCounts.txt.gz), the FPKM values of the genes represented in the fragments (GSE175643_fpkm.txt.gz), and the lengths of the genes from mm10 (GSE175643_geneLength.txt.gz) are publicly available in the NCBI GEO database (GEO:GSE175643)^28^.

The file deposited at Figshare called “Electrophysiological properties of inspiratory preBötzinger complex neurons in neonatal mice (GEO:GSE175643)” presents the electrophysiological profile for each sample preBötC neuron in the data set^29^. The file deposited at Figshare called “Mapping statistics for nucleotide sequences for inspiratory preBötzinger complex neurons in neonatal mice (GEO:GSE175643)” provides a set of statistics from STAR regarding the number of reads, and their lengths, mapped to the mm10 reference genome^29^.

## Technical Validation

We verified average cDNA size and abundance using a Bioanalyzer High Sensitivity kit (Agilent, Santa Clara, CA) and Qubit dsDNA High-sensitivity Assay Kit (Molecular Probes). The raw data of nucleotide sequences along with their corresponding quality scores (FASTQ format) are publicly available in the NCBI GEO database (GEO:GSE175643).

Regarding quality scores, all 20 FastQC reports (two paired-end reads of 10 sample preBötC neurons) showed per sequence quality scores averaging 38–40. None of the sequences were flagged as poor quality.

## Usage Notes

**Fig. 3:**
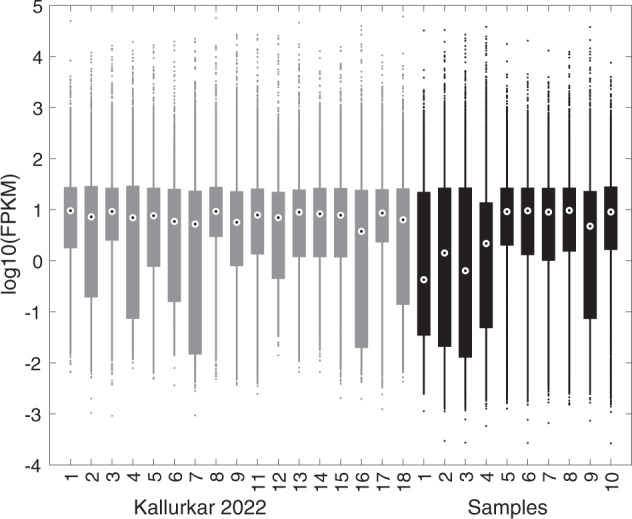
Box plots showing expression levels of the 8,916 genes expressed in 19 transcriptomes of preBötC neurons from either Kallurkar *et al*., 2022 (ref. ^25^) or the 10 samples in the present report. The Kallurkar 2022 data sets are samples 1–9 and 11–18 (grey box plots). The present data sets are samples 1–10 (black box plots). Median log_10_(FPKM) is indicated by bullseye dot in the center of the box plot, which shows the upper and lower quartiles. Dots indicate gene expression levels outside the interquartile range.

**Fig. 4:**
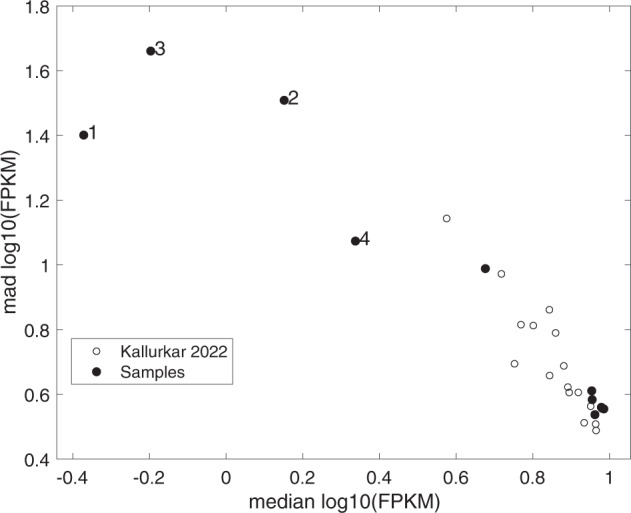
A plot of the median absolute deviation (MAD) of log_10_(FPKM) vs. median log_10_(FPKM) of the 8,916 genes that expressed in at least 19 transcriptomes of preBötC neurons from either Kallurkar *et al*., 2022 (ref. ^25^) (open circles) or the 10 samples in the present report (closed circles). Samples 1–4 with high MAD and low median expression are further indicated with numerals corresponding to sample name.

**Fig. 5:**
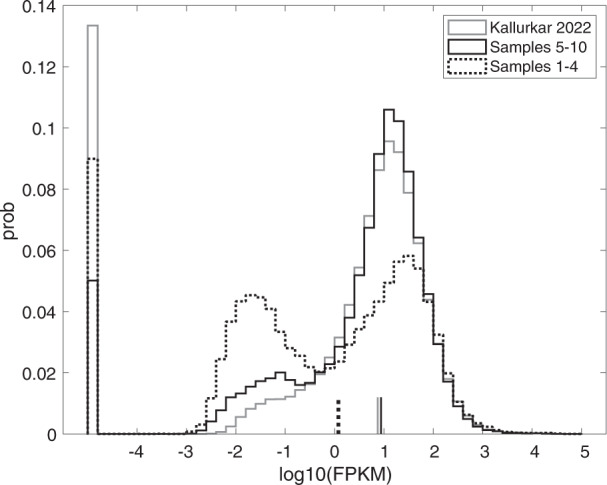
Histogram showing probability of gene expression level for the 8,916 genes expressed in at least 19 transcriptomes of preBötC neurons from either Kallurkar *et al*., 2022 (ref. ^25^) (grey solid line) or the 10 samples in the present report (broken black line for samples 1–4 and solid black line for samples 5–10). The heights of the first bin (0.13, 0.05, and 0.09) correspond to the fraction of mm10 genes not expressed in the Kallurkar 2022 and present data sets.

The transcriptome data described here were collected using techniques identical to the data analyzed in Kallurkar *et al*. *Sci Rep*, 12:2923, 2022, which consisted of 17 Dbx1 preBötC neurons (seven Type-1, nine Type-2, and one Unknown)^25^. Hereafter, we refer to those data as “Kallurkar 2022”. However, the present data were collected and sent for sequencing approximately 1.5 years beforehand so we wished to test for a batch effect that might cause variation between these two data sets. To screen for batch-effect disparity we created boxplots^30^ for each sample summarizing log expression of 8,916 genes with non-zero expression in more than two-thirds of the samples from Kallurkar 2022 and the present data set (i.e., any 19 out of 27 total samples). Those 8,916 genes represent 16% of the 55,367 genes in the mm10 reference genome. Figure 3 shows that the median log_10_(FPKM) hovers at approximately 1.0 across Kallurkar 2022 (grey boxplots, samples 1–9 and 11–18) and the present data (black boxplots, samples 1–10). The length of the boxplots, which span the lower and upper quartiles, are typically one decade (i.e., a factor of 10 in FPKM) but some are as high as three decades (a factor of 1000 in FPKM). However, samples 1–4 in the present data set show median log_10_(FPKM) <0.5 and box length of three decades. Those statistics indicate that samples 1–4 show lower expression levels, for some fraction of the detected genes, and higher variability compared to Kallurkar 2022 and samples 5–10. To explore expression levels and variability further we plotted median log_10_(FPKM) versus the median absolute deviation (MAD) of log_10_(FPKM) (Fig. 4). Samples 1–4 (filled circles with numerals) are positioned in the upper left compared to samples 5–10 (filled circles) and Kallurkar 2022 (open circles), which is again consistent with lower gene expression and higher variability. Finally, we examined a histogram of log_10_(FPKM) for Kallurkar 2022 and the present data (Fig. 5). Kallurkar 2022 (grey line) overlays well with samples 5–10 (solid black line) and the highest peak of samples 1–4 in the present data set (broken black line). However, samples 1–4 exhibit another peak in the distribution at log_10_(FPKM) near –2, indicating a set of low-expressed genes responsible for the higher variability of samples 1–4. Notably, samples 1–4 exhibited the lowest concentration of cDNA according to Qubit quantification, although all four passed the 150 pg/µL criterion^26^. Samples 1–4 have median log_10_(FPKM) that is a factor of 10 lower than samples 5–10 and Kallurkar 2022 (Fig. 5, vertical lines).

We further compared the present data to Kallurkar 2022 by mapping statistics. The fraction of zeros (i.e., the fraction of genes in mm10 that went undetected) was 0.775 in the present data and 0.762 in Kallurkar 2022. The file deposited at Figshare called “Mapping statistics for nucleotide sequences for inspiratory preBötzinger complex neurons in neonatal mice (GEO:GSE175643)” lists the mapping statistics in detail for nucleotide sequences uniquely mapped, multi-mapped, or unmapped to the mm10 mouse reference genome^30^. Mapping statistics for Kallurkar 2022 are associated with ref. ^25^. In brief, 0.85 ± 0.04 represents the fraction of sequences from the present data set mapped uniquely to mm10, 0.05 ± 0.02 represents the multi-mapped fraction, and 0.10 ± 0.03 represents the unmapped fraction. In Kallurkar 2022, 0.56 ± 0.14 represents the fraction of sequences uniquely mapped to mm10, 0.04 ± 0.01 represents the multi-mapped fraction, and 0.16 ± 0.07 represents the unmapped fraction.

We further note that the quality reports for Kallurkar 2022 and the present data return similar scores for “per base sequencing quality”, which was 35 or higher and no sequences flagged as poor quality.

Overall, the log expression boxplots and follow-up analyses rule out major technical variation between the Kallurkar 2022 and the present data sets, with the caveat that a portion of the detected genes in samples 1–4 showed lower overall expression and higher variability compared to samples 5–10 and all the samples in Kallurkar 2022. Nevertheless, the relatively high fraction of uniquely mapped sequences, and low fraction of unmapped sequences, emphasize the quality of the present data set.

Returning to our consideration of the present data set, six inspiratory preBötC neurons were recorded in CD-1 mice. We were only able to differentiate one Type-1 neuron in CD-1 mouse slices; the other five were of Unknown type. We are relatively confident that the Type-1 neuron was excitatory and rhythmogenic on the basis of its membrane properties. However, we cannot be confident about the transmitter type or breathing-related function of the five Unknown neurons from CD-1 wild-type mice.

Of the four Dbx1 preBötC neurons, we detected three Type-1 but only one Type-2. It would be difficult to draw conclusions about differential expression between Type-1 and Type-2 Dbx1 preBötC neurons on the basis of a single Type-2 transcriptome. Given the minimal batch effects (see above), one could analyze the Type-2 transcriptome here with the transcriptomes previously disseminated in Kallurkar *et al*.’s report^25^.

**Fig. 6:**
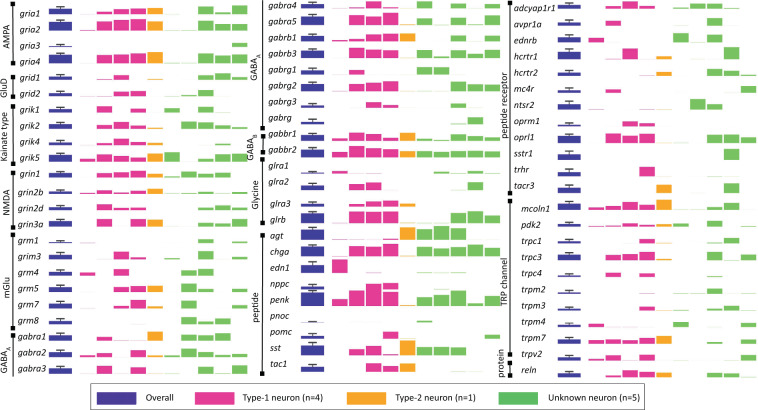
Gene expression levels for ionotropic and metabotropic synaptic receptors, neuropeptides, neuropeptide receptors, transient receptor potential (i.e., Trp) channels, and reelin (rln). The first bar (purple) represents overall gene expression level for all samples (N = 10). Bars to right are grouped by neuron type, in the order of their listing on the NCBI GEO database (#GSE175643): Samples 1–6 are from CD-1 mice; samples 7–10 are from Dbx1;Ai148 mice. Type-1 neurons (pink) correspond to samples 4–7; the Type-2 neuron (orange) corresponds to sample 1; and Unknown neurons (green) correspond to samples 2, 3, 8–10. The height of the bar represents log_2_(mean FPKM) value; error bars show standard deviation across all samples (purple bars only).

**Fig. 7:**
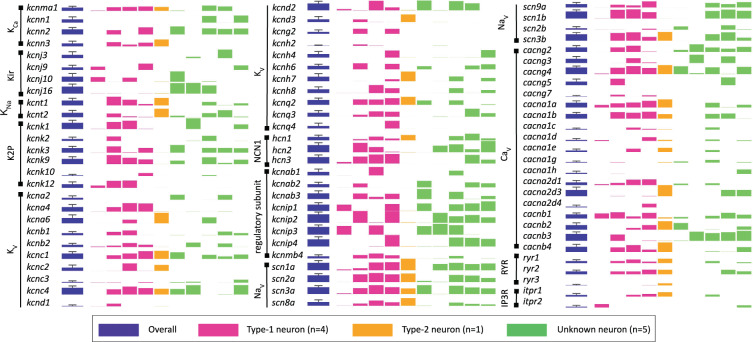
Gene expression for voltage-dependent ion channels, regulatory subunits, and intracellular receptors. The first bar (purple) represents overall gene expression level for all samples (N = 10). Bars to the right are grouped by neuron type, in the order of their listing on the NCBI GEO database (#GSE175643): Samples 1–6 are from CD-1 mice; samples 7–10 are from Dbx1;Ai148 mice. Type-1 neurons (pink) correspond to samples 4–7; the Type-2 neuron (orange) corresponds to sample 1; and Unknown neurons (green) correspond to samples 2, 3, 8–10. The height of the bar represents log_2_(mean FPKM) value; error bars show standard deviation across all samples (purple bars only).

In Figs. 6 and 7, we highlight the expression levels of genes that are of common interest to respiratory physiologists. The bars are color coded to indicate Type-1 (magenta), Type-2 (orange), and Unknown (green). The height of the bars corresponds to the log_2_(FPKM) expression level for each gene. Purple bars show the mean and SD across the entire sample. Figure 6 includes ionotropic and metabotropic synaptic receptors, neuropeptides, neuropeptide receptors, transient receptor potential (i.e., Trp) channels, and reelin (*rln*). Figure 7 presents voltage-dependent ion channels, regulatory subunits, and intracellular receptors.

The neurons whose transcriptomes we present here are the same population of Dbx1-derived and non-Dbx1-derived neurons described by Hayes and colleagues^31^. However, in that study the preBötC neurons were not intracellularly recorded. One could compare the present preBötC neuron transcriptomes to those in Hayes *et al*. keeping in mind that the physiological phenotypes of the latter neurons were not identified^31^.

## Data Availability

The custom R scripts written to process the raw fragments counts are freely available (https://github.com/prajkta9/bioinformatics-scRNA-seq).
